# Application of virtual planning to maxillofacial reconstruction with an implant prosthesis: a case report

**DOI:** 10.1186/s40729-025-00600-2

**Published:** 2025-02-24

**Authors:** Yutaro Oyamada, Hiroyuki Yamada, Ikuya Miyamoto, Hisatomo Kondo

**Affiliations:** 1 Division of Fixed Prosthodontics and Oral Implantology, Department of Prosthodontics, School of Dentistry, Iwate Medical University, 19-1 Uchimaru, Morioka, Iwate 020-8505 Japan; 2 Division of Oral and Maxillofacial Surgery, Department of Reconstructive Oral and Maxillofacial Surgery, School of Dentistry, Iwate Medical University, Iwate, Japan; 3 Department of Oral Diagnosis and Medicine, Faculty and Graduate School of Dental Medicine, Hokkaido University, Sapporo, Japan; 4 Department of Fixed Prosthodontics and Oral Implantology, School of Dentistry, Aichi Gakuin University, Aichi, Japan

**Keywords:** Virtual planning, Maxillofacial reconstruction, Implants, Intraoral scanner, 3D printer, Costumed titanium mesh tray

## Abstract

**Background:**

A case of postoperative mandibular defects was successfully managed using an intraoral scanner and computer-aided design/computer-aided manufacturing (CAD/CAM) technology, facilitating jaw reconstruction and functional restoration with implants for a critical mandibular defect.

**Case presentation:**

The intraoral scanner was used to scan the maxilla and mandible, and occlusal scans were acquired. The obtained data were imported to CAD/CAM software to design the virtual teeth. Digital Imaging and Communications in Medicine data of preoperative cone-beam computed tomography images were converted to three-dimensional (3D) data using specialized software to examine the mandibular bone volume and modify the jawbone morphology. All data were superimposed on the implant simulation software, and jawbone morphology was modified considering the implant placement position. The finalized jawbone 3D data were printed using a 3D printer. Then, a titanium mesh tray was fabricated on the 3D printed model. Subsequently, iliac cancellous bone grafting using a titanium mesh tray and implant treatment were performed.

**Conclusions:**

The application of digital technology helped visualize the final image of the treatment result and collaborate closely with the oral surgeon from the pre-reconstruction stage. This technique allows mandible reconstruction after considering the implant placement based on the ideal prosthesis.

## Background

The application of implants to maxillofacial reconstruction of postoperative mandibular defects, such as after cancer resection, allows recovery of masticatory function [1]. However, maxillofacial reconstruction using only the tibia, fibula, or titanium plates is unlikely to result in correct implant positioning for an ideal prosthesis. Therefore, mandible reconstruction using a custom-made titanium mesh (cTiMesh) tray is performed using a mirroring model of the preoperative or healthy jawbone, with particulate cancellous bone and marrow (PCBM) achieving better results [2–4]. Using a mirroring model or a preoperative jawbone to achieve ideal reconstruction may be difficult depending on the fixation position of the titanium plate after mandibulectomy and changes in the oral environment, including the temporomandibular joint and opposing dentition after a prolonged period of defect [5].

Here, we report a case of mandibular bone defects in which implant placement was performed using an intraoral scanner, computer-aided design/computed-aided manufacturing (CAD/CAM) software, and a three-dimensional (3D) printer to create a cTiMesh tray and a jawbone model that served as a reference for the implant position based on the design of the ideal dental arch for mandibular bone reconstruction.

## Case presentation

### Patient

**Fig. 1:**
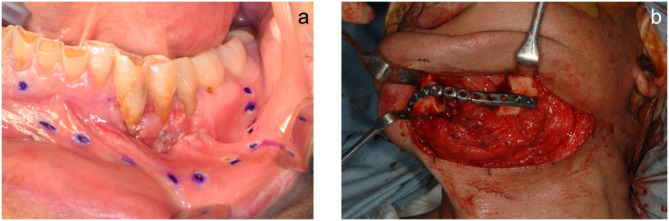
Mandibulectomy and mandible reconstruction using a titanium plate. **a**. Preoperative intraoral photo image. **b**. Intraoral photograph during surgery

**Fig. 2:**
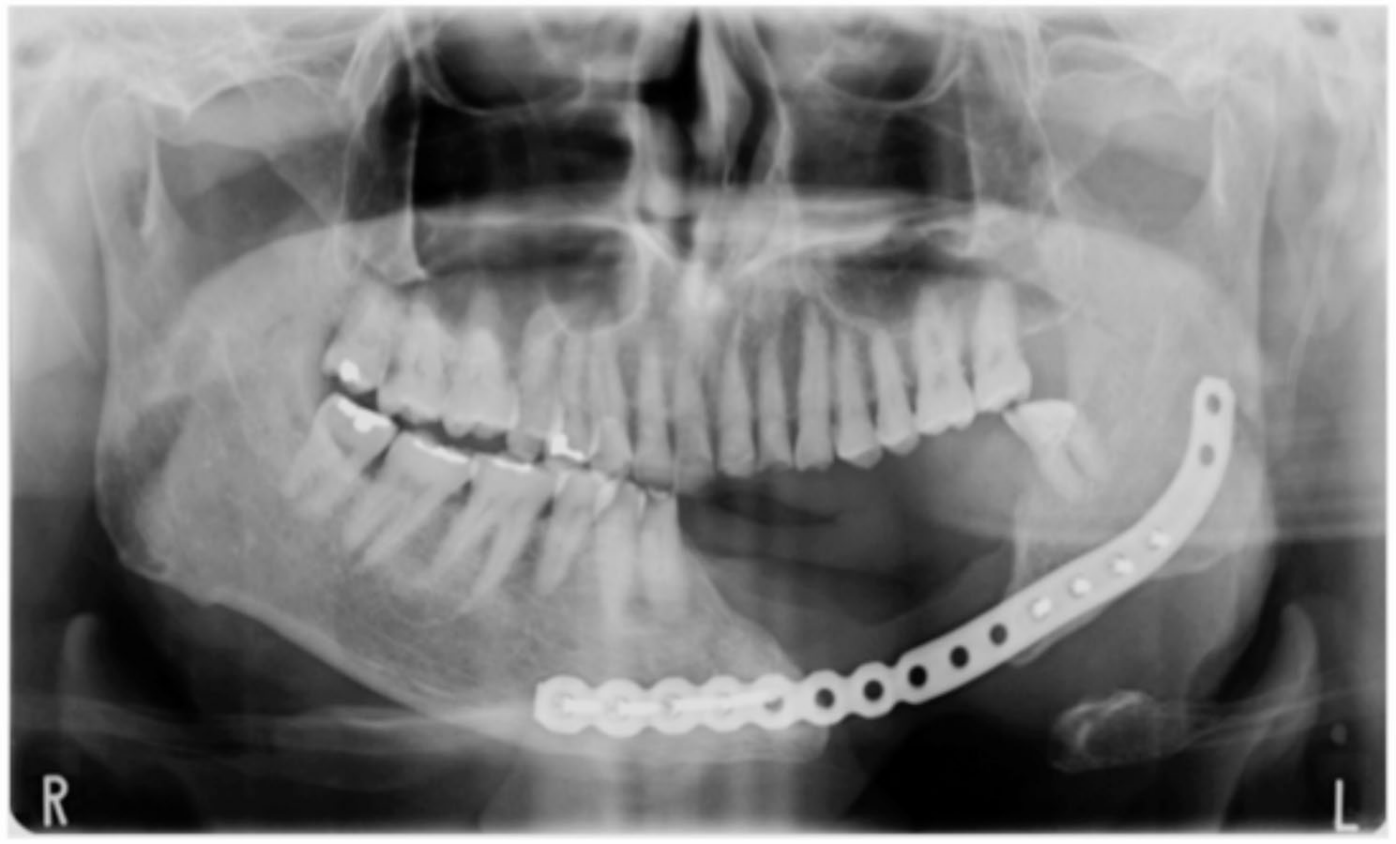
Postoperative panoramic image

A 70-year-old man underwent left-sided segmental mandibulectomy and titanium plate fixation caused by gingival cancer of the mandible in April 2016 (Figs. 1 and 2). He has had an uneventful course after hospital discharge without recurrence of cancer for more than 2 years. Her main symptoms were masticatory disturbance and cosmetic disturbance due to serious mandibular defect. The examinations were performed with the patient’s consent. Cone-beam computed tomography revealed that the mandible, extending from the right lateral incisor to the left second molar, had undergone resection and reconstruction with a titanium plate. Additionally, there was no evidence of tumor recurrence in the mandible. Due to insufficient bone volume at the site for implant placement, a substantial bone graft was necessary. Blood tests and periodontal exam showed no abnormalities. The cast diagnosis on an articulator mounted by maxillomandibular relationship with an occlusal rim was difficult since the mandibular defects lacked keratinized mucosa. After consultation and examination, we decided to plan implant treatment after PCBM grafting using a cTiMesh tray to restore the function of the mandibular defect.

### Mandibular reconstruction based on the virtual planning

**Fig. 3:**
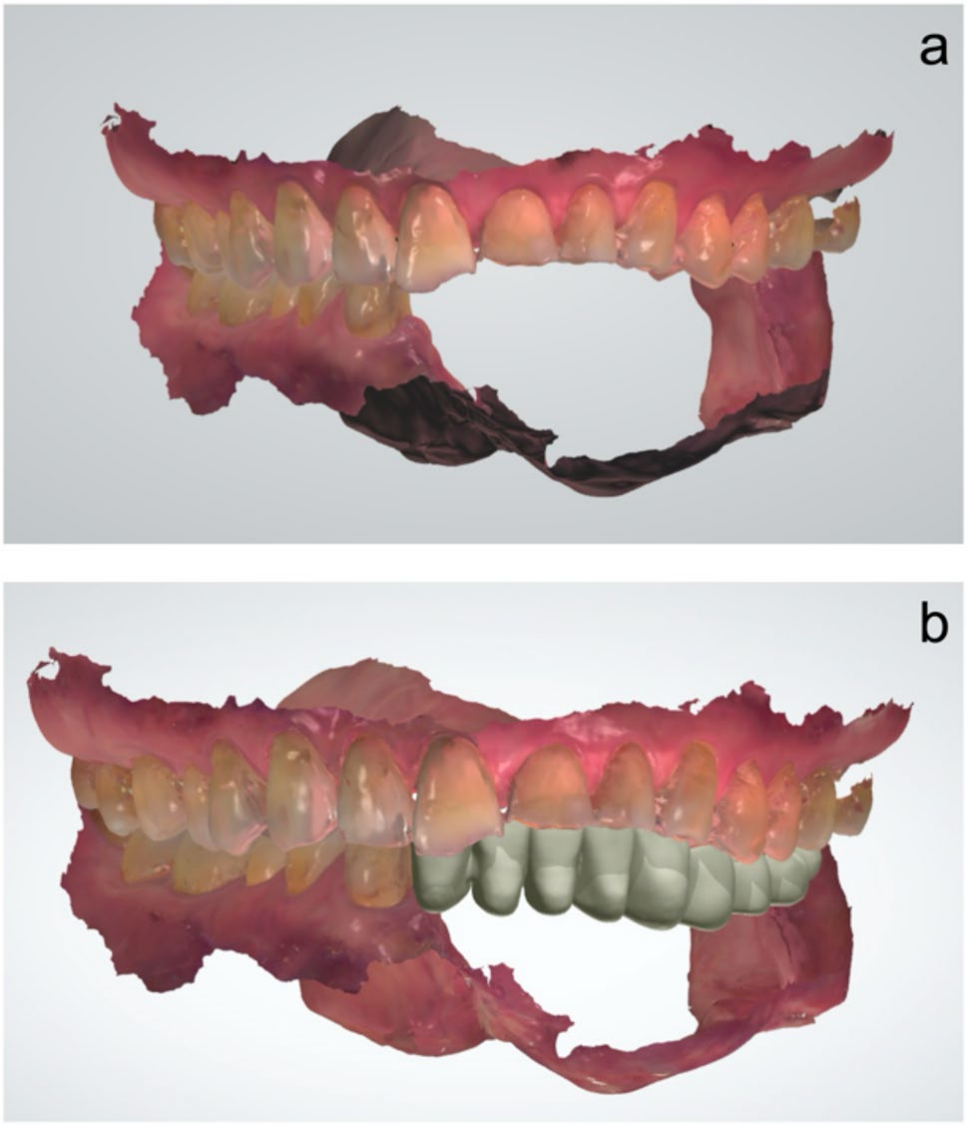
Design of the virtual teeth on CAD/CAM software. **a**. Scans of the maxilla and mandible. **b**. Design of the virtual teeth

An intraoral scanner (TRIOS3, 3Shape) was used to scan the maxilla and mandible as well as the occlusion. During the scanning of the mandibular defect area, the mucosa was stretched to ensure optimal visualization, and the scan was performed to achieve continuity with the scan images of the natural teeth. The scan data were exported in the DCM format (Fig. 3a). The edentulous area was scanned accurately with no image defects caused by tongue movements or the buccal mucosa.

**Fig. 4:**
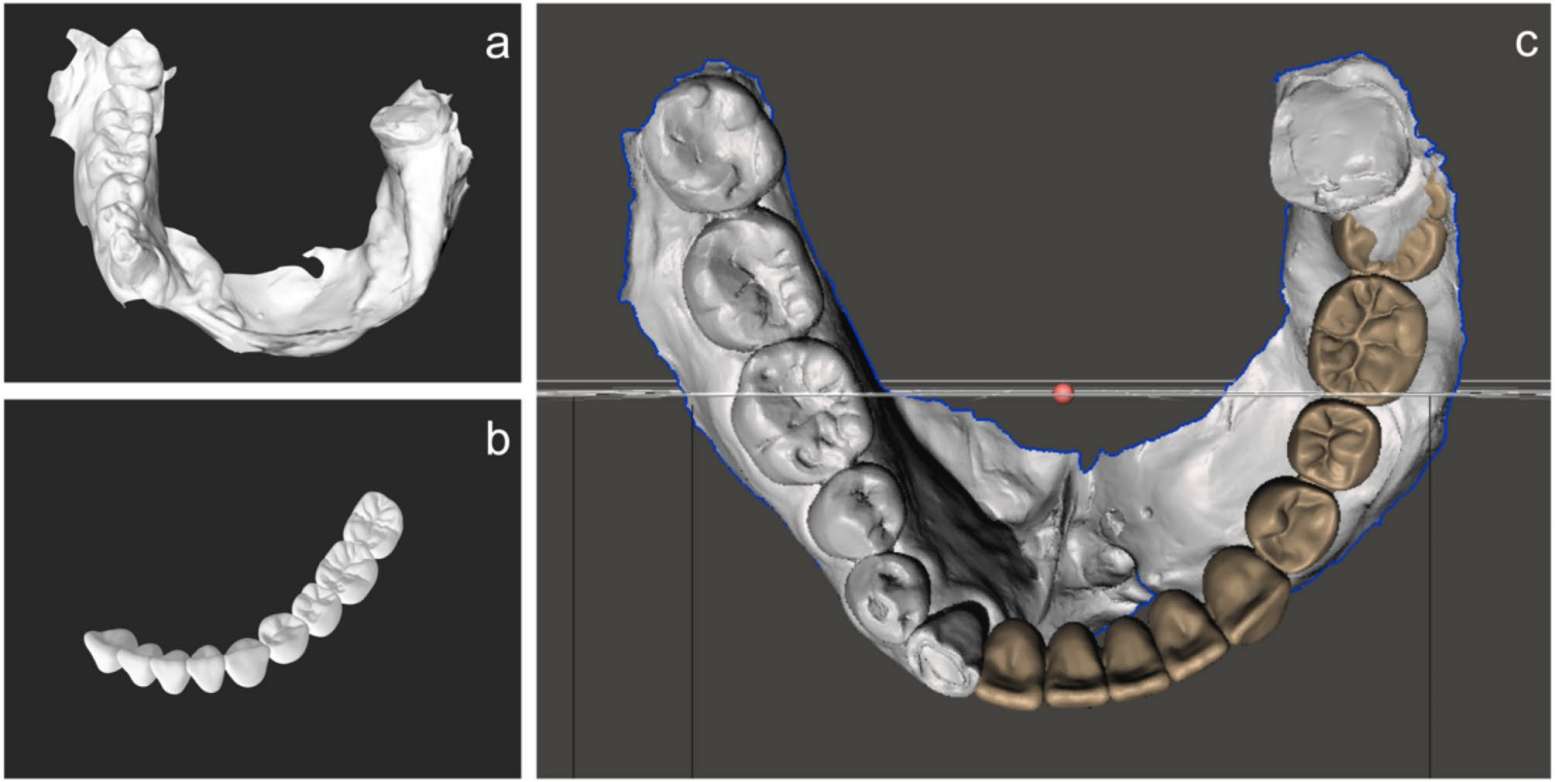
STL file after designing the virtual teeth. **a**. STL file of the mandible. **b**. STL file of the virtual teeth. **c**. Combined STL file

The obtained data were imported into CAD/CAM software (Dental system 2017, 3Shape). The virtual teeth were designed (Fig. 3b) and exported in the Standard Triangle Language (STL) format. The plan was to extract the left mandibular third molar because of malpositioning. Since the STL files of the virtual teeth and mandible were exported separately, they were combined using CAD/CAM software (Meshmixer, Autodesk) (Fig. 4a-c).

**Fig. 5:**
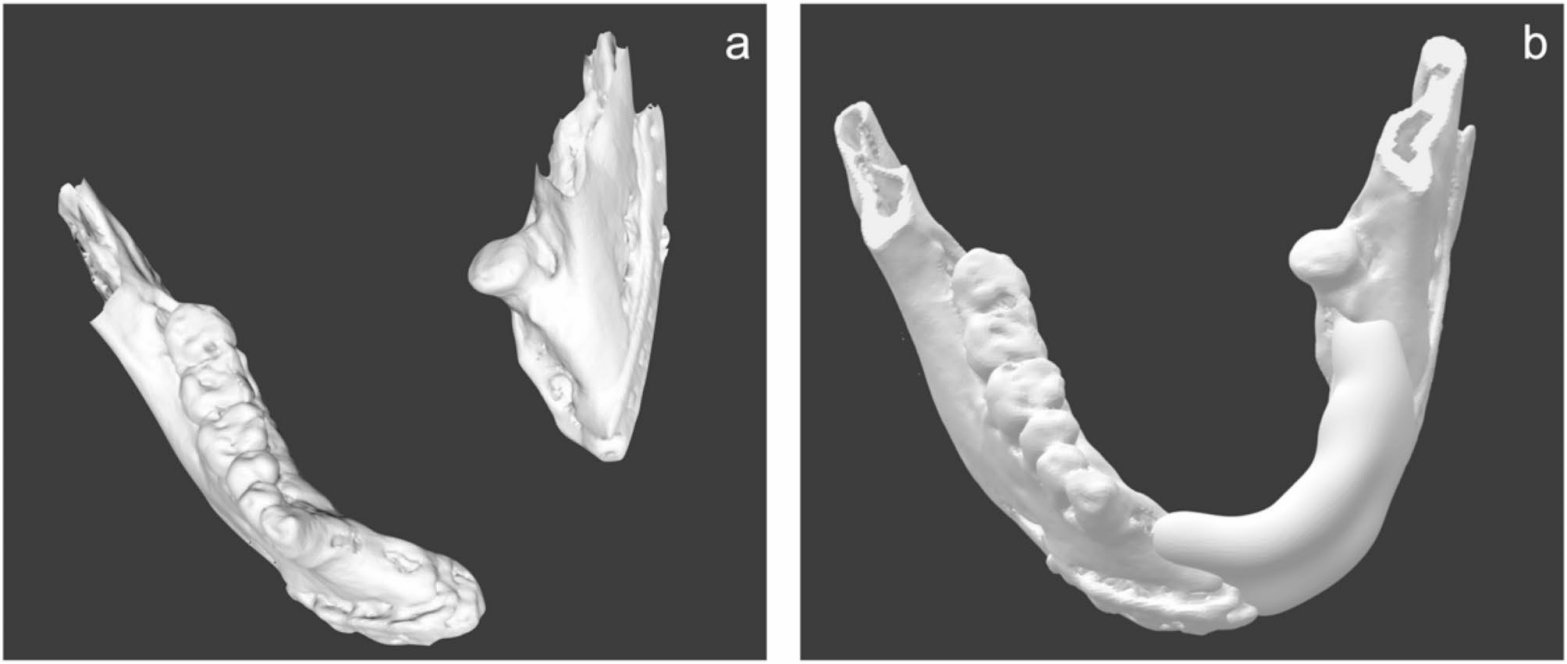
Modification of the mandible on CAD/CAM software. **a**. STL file of the mandible. **b**. STL file of the modified mandible

Surface rendering of the mandible was performed from Digital Imaging and Communications in Medicine (DICOM) data using medical imaging software (OsiriX, Pixmeo) and exported in the STL format (Fig. 5a). The exported STL data of mandible were imported into CAD software (Geomagic Freeform, 3D Systems) for morphological modification to ensure suitability for ideal implant placement (Fig. 5b). The morphology of the contralateral mandible was used as a reference during this process.

**Fig. 6:**
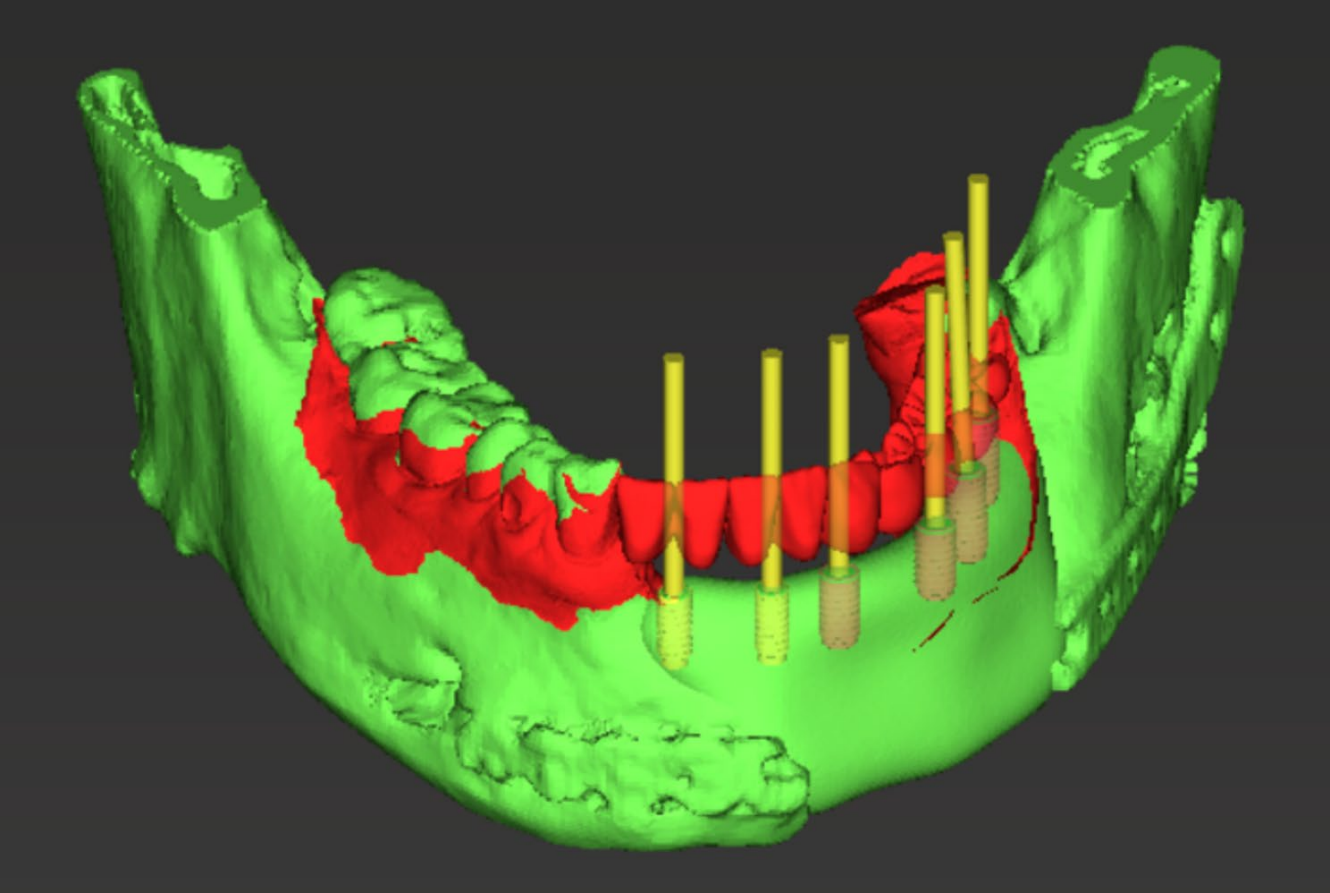
3D image on the virtual implant planning system and position of implant placement

The DICOM and STL files of the virtual teeth and modified mandible were imported into a virtual implant planning system (CoDiagnostiX, Straumann AG) to examine the implant placement position (Fig. 6). The mandibular morphology was modified according to the position of implant placement in CAD software as necessary.

**Fig. 7:**
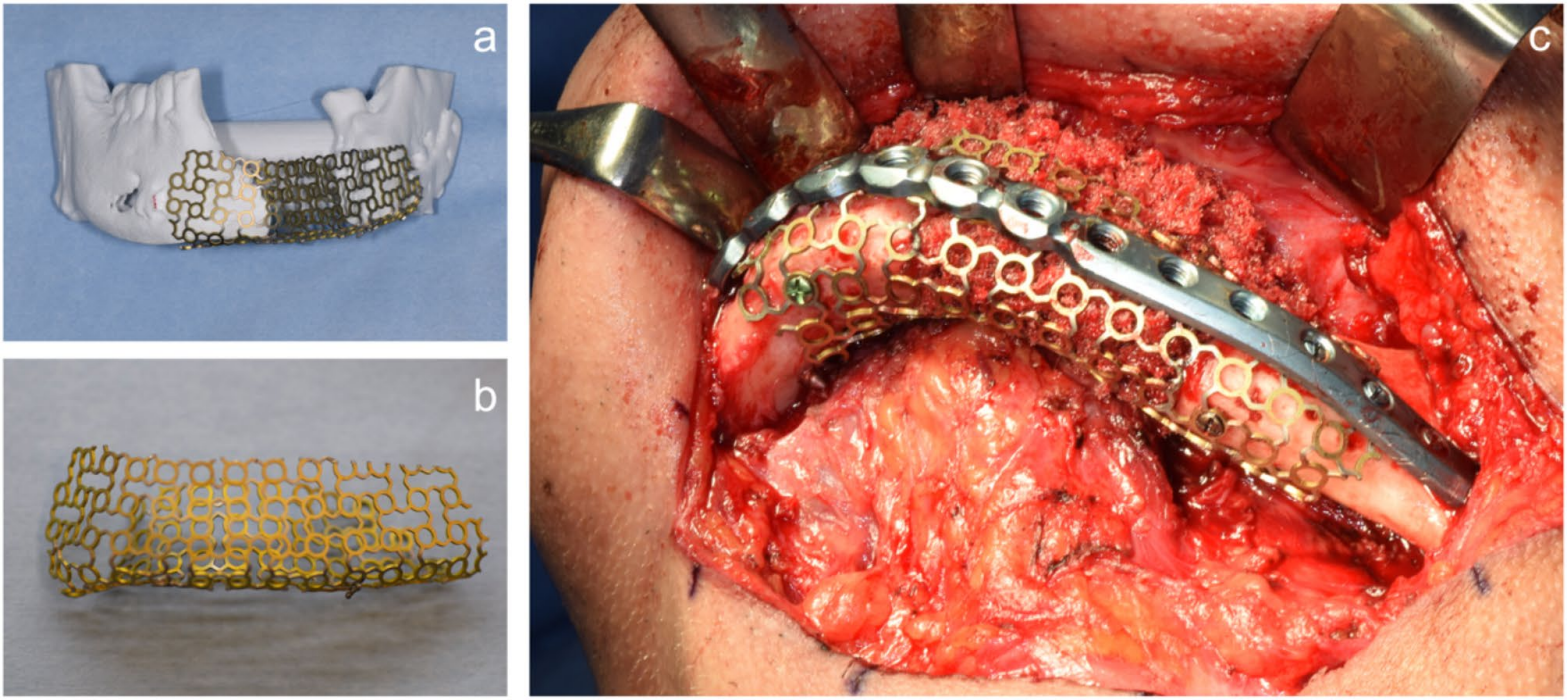
PCBM grafting using the cTiMesh tray. **a**. 3D-printed cast. **b**. cTiMesh tray. **c**. Intraoral photograph during surgery

The STL file of the modified mandible was exported in the STL format and built as 3D printed cast using a 3D printer (ZPrinter 450, 3D Systems) (Fig. 7a). A titanium mesh (Gold 3D mesh, Stryker) was pre-bent and weld on the 3D printed cast to fabricate the cTiMesh tray (Fig. 7b).

PCBM grafting using the cTiMesh tray was performed under general anaesthesia in May 2018 (Fig. 7c). Following the administration of infiltration aesthesia, an extraoral incision was made to minimize the risk of postoperative wound dehiscence. The soft tissues, including muscles, were carefully dissected, and the titanium plate previously used in the initial mandibular reconstruction was temporarily removed. cTiMesh tray was trial-fitted, and its suitability was confirmed. Simultaneously, cancellous bone was harvested from the iliac crest by orthopaedic surgeons. The harvested cancellous bone was packed into cTiMesh tray, which was then securely fixed to the mandible. Due to the extensive bone graft, the previously removed titanium plate was reattached to reduce the risk of titanium mesh tray failure. After confirming the stability of the fixation, the procedure was concluded with meticulous to ensure proper wound closure. Following a healing period of approximately four months, palatal mucosal grafting was conducted in September 2018 to address the deficiency of keratinized mucosa, which is critical for ensuring peri-implant tissue health and stability.

### Implant treatment

**Fig. 8:**
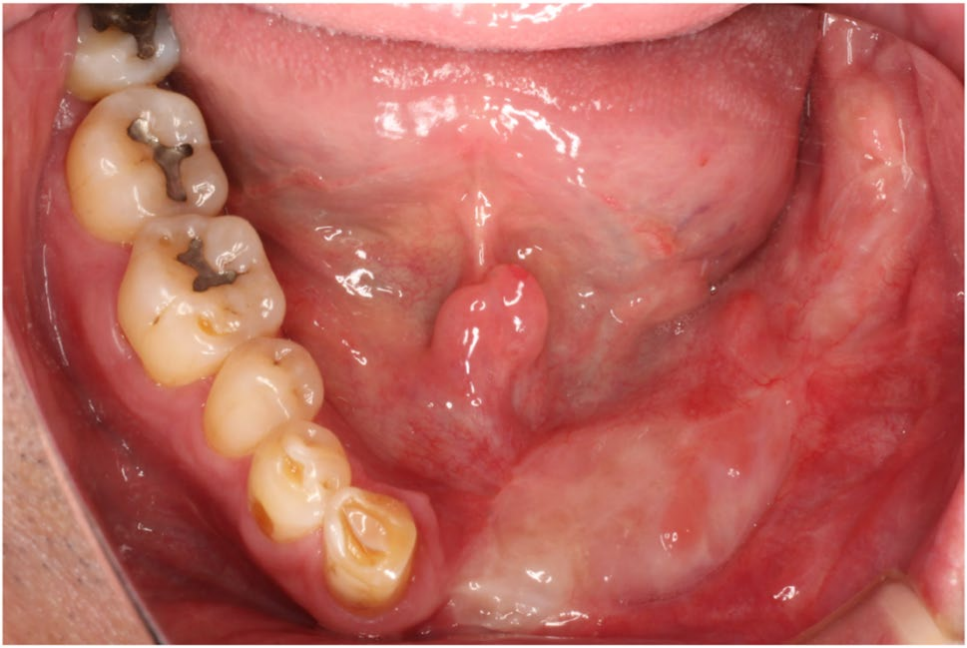
Intraoral photograph before implant placement

**Fig. 9:**
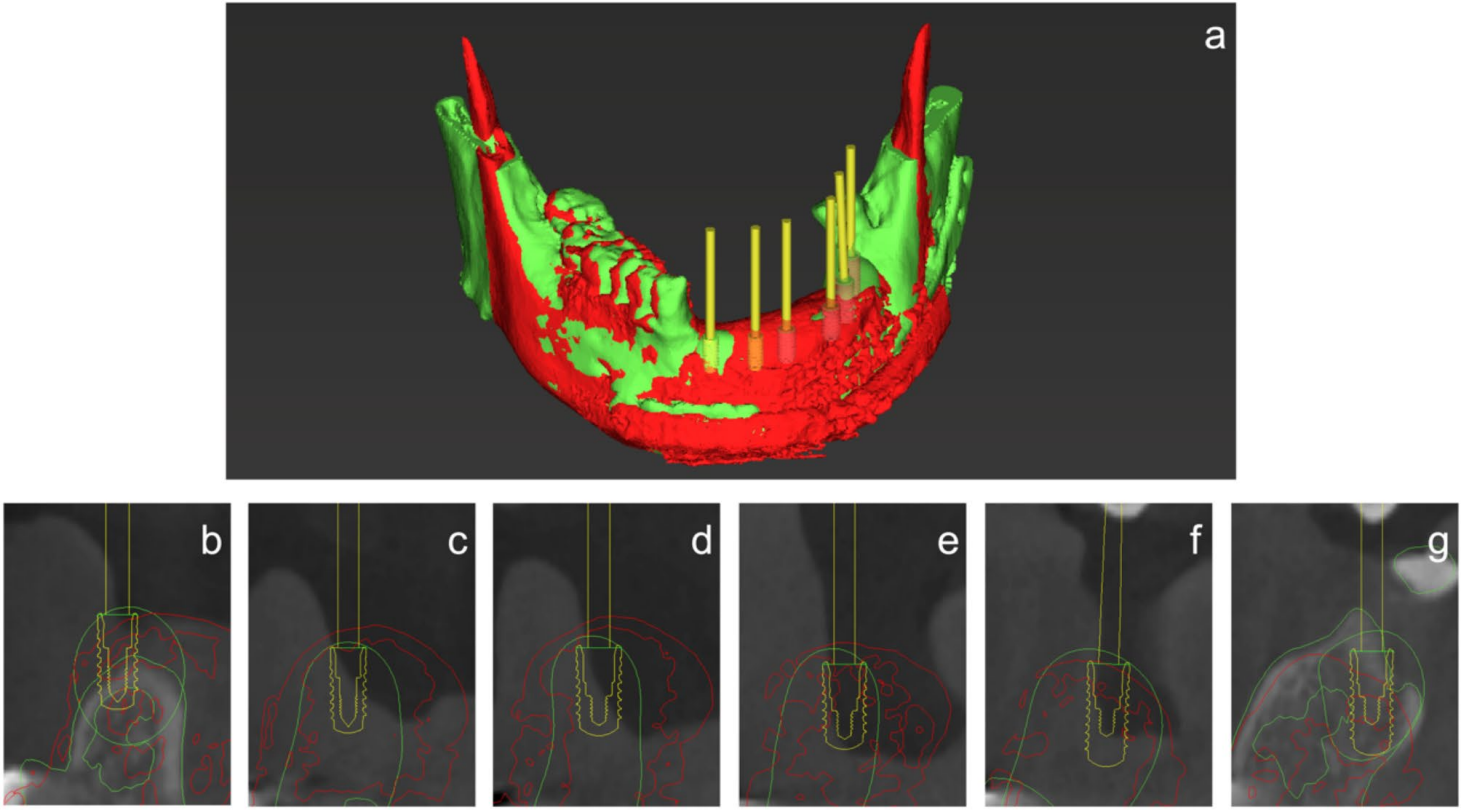
3D image on the virtual implant planning system and position of implant placement. **a**. Superimposition of the 3D image of the preoperative modified STL file of the mandible (green) and postoperative image of the mandible (red). **b**. 42. **c**. 32. **d**. 33. **e**. 35. **f**. 36. **g**. 37

**Fig. 10:**
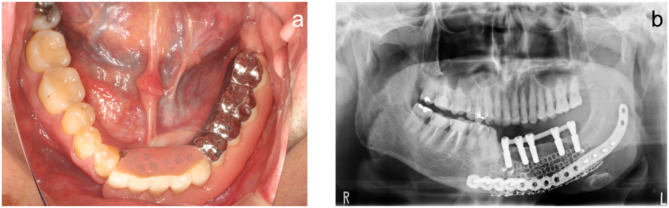
Maxillofacial prosthesis placement. **a**. Intraoral photograph. **b**. Panoramic image

**Fig. 11:**
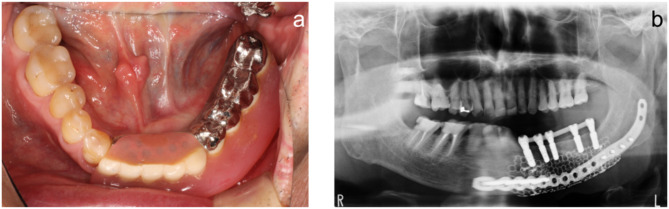
Maxillofacial prosthesis placement after 3 years. **a**. Intraoral photograph. **b**. Panoramic image

**Fig. 12:**
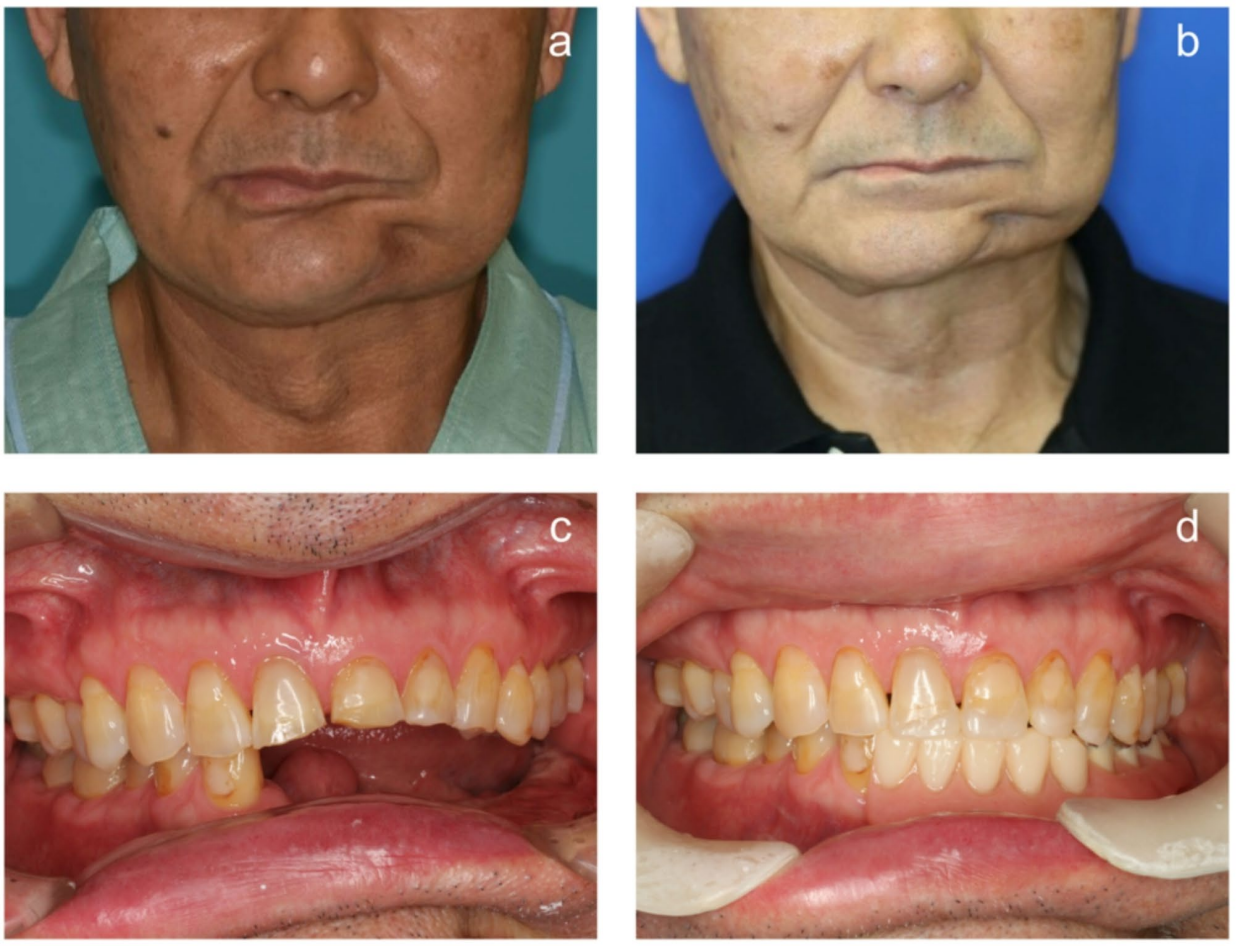
Preoperative and postoperative facial and intraoral images. **a**. Preoperative facial view. **b**. Postoperative facial view. **c**. Preoperative intraoral view. **d**. Postoperative intraoral view

The bone and mucosa showed excellent healing in November 2018 (Fig. 8). The mandibular morphology was evaluated on the virtual implant planning system before and after bone grafting, and the reconstruction was found to be almost as planned (Fig. 9a-g). Prior to the implant placement surgery, another simulation was conducted to reassess the implant positions based on the healing progress of the augmented bone and surrounding tissues. The patient underwent the first implant placement surgery (#42: Straumann BL NC 3.3 × 10 mm, #32: Straumann BL NC 3.3 × 10 mm, #33: Straumann BL NC 3.3 × 10 mm, #35: Straumann BL RC 4.1 × 10 mm, #36: Straumann BL RC 4.1 × 10 mm, Straumann AG) in February 2019, followed by a second surgery in September 2019. Subsequently, a fixed implant provisional restoration on multiunit abutment (#42: Straumann SRA 4.6 × 4 mm, #32: Straumann SRA 4.6 × 2.5 mm, #33: Straumann SRA 4.6 × 2.5 mm, #35: Straumann SRA 4.6 × 4 mm, #36: Straumann SRA 4.6 × 4 mm, Straumann AG) was placed after open tray impression. However, issues such as cheek mucosa biting and poor cleanability were observed. Cheek mucosa biting was resolved by adding auto-polymerized resin to the buccal side of the provisional restoration, increasing its volume. Therefore, the final restoration was decided to be a removable prosthetic device. The open tray impression performed using custom tray. Considering the occlusal forces due to the opposing natural teeth, a bar attachment was selected for the prosthesis [6]. The removable maxillofacial prosthesis with a bar attachment was placed in August 2020 (Fig. 10a, b). The definitive denture did not cause mucosal erosion and functioned effectively without any complications in the oral cavity. The results of the oral function assessment conducted using the glucose sensor (GLUCO SENSOR GS-II, GC Corporation, Tokyo) and pressure-sensitive film (Dental Prescale II, Fuji Film CO.), both before and after the procedure, are presented in Table 1. A comparison of preoperative and postoperative data demonstrates an improvement in oral function following the procedure.Three years have passed since the prostheses was placed, and damage to the superstructure, mucosal inflammation surrounding the implant, or implant loss has not occurred (Fig. 11a, b). In conjunction with mandibular reconstruction, the flange of the removable prosthesis restored the fullness of the left lower lip, improving the facial appearance compared to the preoperative state. Furthermore, prosthetic treatment of the defect area, combined with direct restoration of natural teeth, including the anterior teeth, facilitated the recovery of both functionality and a certain degree of aesthetics. (Fig. 12). After the placement of the prosthesis, the patient expressed satisfaction both functionally and aesthetically.

**Table 1:** Comparative assessment of preoperative and postoperative oral function

	Bite force (*N*)	Occlusal Contact Area (mm^2^)	Masticatory performance (mg/dL)
Preoperative oral function	450.4	10.6	159
Postoperative oral function	691.1	24.7	191

## Discussion

Accurate implant placement is difficult when guidance stents are not placed in the reconstructed jawbones and anatomical landmarks are lost [7]. Therefore, planning for maxillofacial reconstruction by working backward from the implant placement is an effective method. Maxillofacial reconstruction with implant prostheses after bone grafting using a 3D-printed cast, cTiMesh tray, and PCBM graft have often been reported [2–4]. However, changes in the oral environment, including the temporomandibular joint and opposing dentition due to the fixation position of the titanium plate after mandibulectomy and prolonged defects, may make it challenging to provide the ideal dental implant treatment through reconstruction using reversed 3D data of a preoperative or healthy jaw [5]. Therefore, an implant placement plan is required using the reconstituted data of the oral environment. In the present case, this problem could be solved using the existing digital technology. Scanning with an intraoral scanner helped perform the conventional steps of impression making and occlusal registration simultaneously. The conventional method using an occlusion rim would have yielded unstable results in the present case because of the extensive crest resorption and no keratinized mucosa, which are likely to cause errors.

Intraoperatively, the reduction in operation time and invasiveness by preparing the titanium mesh tray in advance is desirable and leads to a successful surgery [2–5]. Moreover, implant placement is difficult without adequate bone grafting.

In treating postoperative defects caused by jaw cysts, as in this case, multidisciplinary cooperation is necessary for successful treatment, including of oral surgeons, plastic surgeons, otolaryngologists, prosthodontists, dental technicians, speech–language pathologists, and dental hygienists for postoperative maintenance. All personnel should have relevant expertise; however, this is difficult to achieve. Moreover, the treatment workflow is difficult to visualize. Digital technology helps share images and opinions and clarify and unify the goals of complicated maxillofacial prosthodontic treatment. In the future, digital technology should be used in the sequence of treatment planning, explanation, surgery, and prosthetic treatment with further developed technology, such as extended reality [8].

A limitation of this method is the lack of sufficient predictability for soft tissue grafts. For long-term stability of implant treatment, an appropriate mucosal thickness without excess fatty tissue is desirable to prevent inflammatory reactions around the implant and achieve an appropriate oral vestibule [9]. Although the mucosal volume is possible to examine quantitatively based on digital data, the spreadability and attachment site of mucosa after mucograft placement are challenging to predict because virtual simulation is impossible to represent a flexible tissue. Therefore, the shape and type of prostheses are necessary to modify or change, as required as in this case. Furthermore, to establish this technique as the standard of care, it is desirable to increase public funding for the materials and techniques used in maxillofacial reconstruction treatment.

This method describes prosthetically driven maxillofacial reconstruction, a surgical plan based on the definitive implant prosthesis using existing digital technology.

## Conclusion

The application of existing digital technologies, including intraoral scanners, various CAD/CAM software, and 3D printers, enables the fabrication of highly predictable maxillofacial implant prosthetics for extensive maxillofacial defects.

### Previous presentation

This case report was presented at Academy of Osseointegration Annual Meeting 2023 in Arizona in March 2023.

## Data Availability

No datasets were generated or analysed during the current study.

## Abbreviations

cTiMesh: Custom-made titanium mesh
